# Quality improvement in neurosurgery: A systematic review

**DOI:** 10.3892/mi.2025.222

**Published:** 2025-02-24

**Authors:** Mohamed M. Madan, Ahmed M. Alshereiqi, Noor M. Abdulla, Maryam Albreiki, Tariq Al-Saadi

**Affiliations:** 1 College of Medicine and Health Sciences, National University of Science and Technology, Sohar 329, Sultanate of Oman; 2 Medical School, University of Nicosia, 8025 Nicosia, Cyprus; 3 Oman Medical Speciality Board, Saham, Mukhaleef 319, Sultanate of Oman; 4 Department of Neurosurgery, Cedars-Sinai Medical Centre, Los Angeles, CA 90048, USA

**Keywords:** quality improvement, neurosurgery, patient safety, patient care, spine surgery, cranial surgery, ERAS, protocols

## Abstract

Quality improvement (QI) is crucial for advancing patient care and safety in surgical practices. Despite the presence of numerous systematic reviews on various types of surgeries, no current QI systematic review for neurosurgery is available, at least to the best of our knowledge. The present study thus aimed to explore existing QI frameworks, interventions and outcome measures, which are used to enhance patient care and efficiency in neurosurgery. For this purpose, a systematic review was conducted by identifying 75 articles using key words, such as ‘Quality’, ‘Control’, ‘Improvement’, ‘Neurosurgical’ and ‘Neurosurgery’ across various databases, including PubMed, Google Scholar, Scopus, Wiley, ScienceDirect and Microsoft Academic. Each article was assessed based on inclusion and exclusion criteria, without a time limit for selection. The analysis of the 75 publications revealed an uneven distribution across neurosurgical fields: Adult neurosurgery (70.5%), spine surgery (22.5%), pediatric neurosurgery (4%) and neuro-oncology (3%). This pattern was reflected in the patient distribution (n=621,293), with 87.07% involved in spine surgery QI initiatives. Cranial-only and combined cranial and spinal studies accounted for only 0.21% of patients. QI interventions included mainly new protocols (18.67%), ERAS (17.33%), data analysis (16%), modified checklists (14.67%) and new sterilization devices (13.3%). By contrast, only a limited number of articles addressed the effectiveness of new technology, prediction models, incident reporting and staff education. On the whole, the QI studies enhanced neurosurgical care, focusing mainly on adult neurosurgery and targeting specifically spinal cases. The main interventions included new protocols, ERAS, data analysis and checklists. Further research is required to address QI initiatives in cranial surgery and evaluate the effectiveness of less commonly used methods, such as new technologies and predictive models.

## Introduction

Quality improvement (QI) is a critical aspect of advancing patient care, enhancing outcomes and ensuring the highest standards of safety and efficiency in surgical practices (1-4). Neurosurgery, with its inherent complexities and high risks, demands attention to detail and a persistent pursuit of excellence. Severe perioperative complications in neurosurgical patients can lead to considerable harm, morbidity, permanent disability, or even mortality. The recovery period following such procedures is frequently complex, requiring extended rehabilitation and the use of expensive specialized resources (2-5). The field has seen notable advancements over the years, driven by innovative surgical techniques, cutting-edge technology and a deeper understanding of neurological conditions (3). However, the complexity and risks associated with neurosurgical procedures require a robust framework for continuous QI. This involves systematically identifying areas for enhancement, implementing evidence-based interventions, and rigorously evaluating outcomes to ensure sustained improvements (5). In this context, QI initiatives aim to reduce complications, optimize patient recovery, and enhance overall surgical success rates, thereby elevating the standard of care for patients (6).

Despite the presence of numerous systematic reviews on various types of surgeries, such as otolaryngology-head and neck surgeries, cleft palate surgeries and laparoscopic surgeries (7-9), current systematic reviews for QI dedicated to neurosurgery have not been established, at least to the best of our knowledge. The present systematic review thus aimed to explore existing evidence, gaps in current practices and standardized QI measures utilized in neurosurgery.

## Data and methods

**Figure 1:**
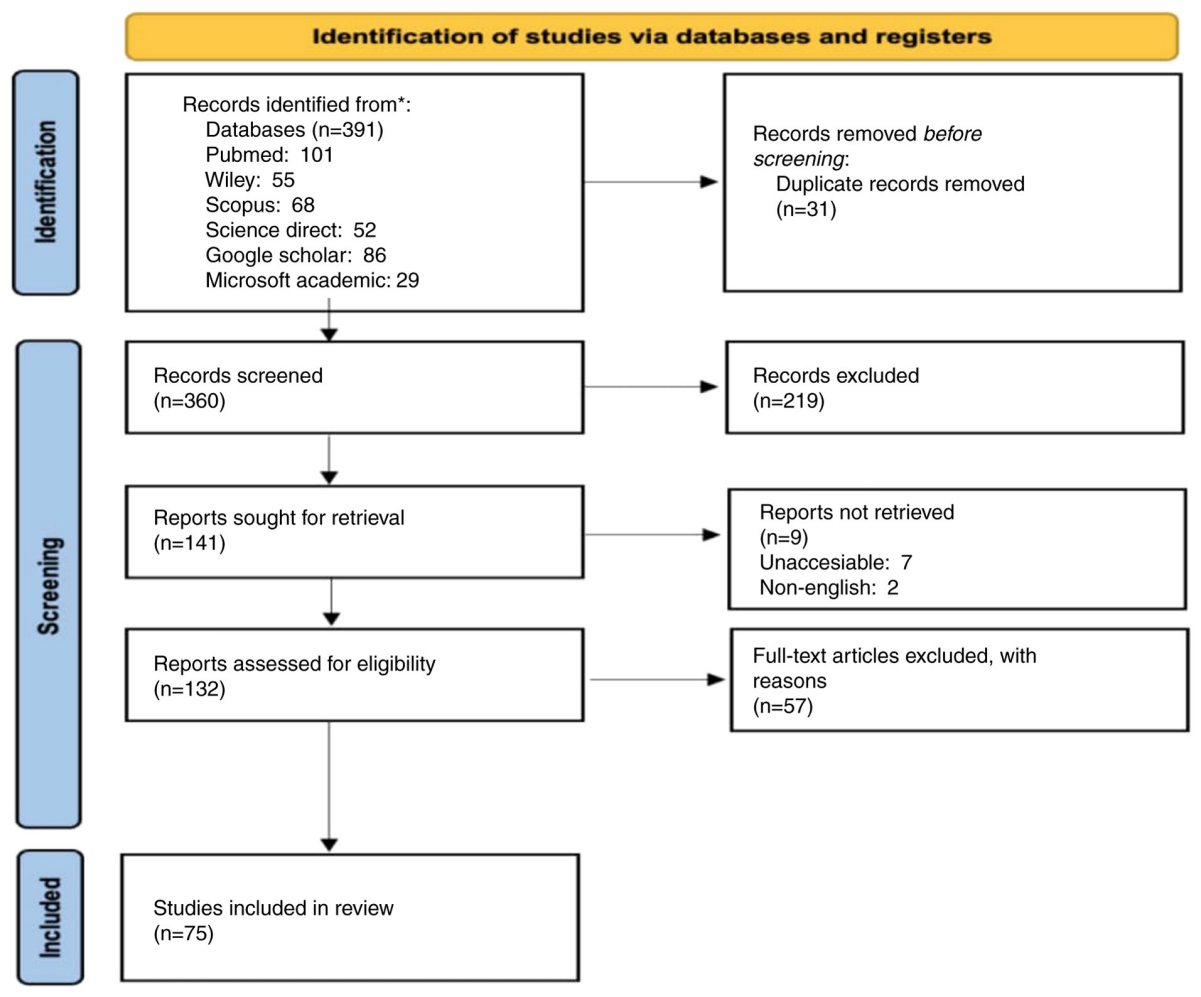
PRISMA flow diagram illustrating the process of article inclusion and exclusion for the present systematic review.

For the present systematic review, articles were selected based on a set of predetermined key words, including ‘Quality’, ‘Control’, ‘Improvement’, ‘Neurosurgical’ and ‘Neurosurgery’. These key words were strategically selected to ensure a comprehensive search and capture all relevant studies within the scope of neurosurgical quality control and improvement. In order to gather the scientific publications, the key words were applied across six major internet search databases: PubMed, Google Scholar, Scopus, Wiley, ScienceDirect and Microsoft Academic. These databases were selected due to their extensive repositories and relevance to the field. Initially, a total of 391 scientific publications were retrieved from these databases. During the initial review, 31 articles were found to be duplicates and were subsequently removed, leaving a total of 360 unique papers for further screening (Fig. 1). The screening process was meticulous and involved a detailed examination of the relevance of each article to the inclusion and exclusion criteria set by the research team. In order to maintain the meticulousness of the screening process at a high level, all reviews and studies that tended to provide information on new interventions to improve the quality of services in neurosurgery were included; articles that discussed non-neurosurgical interventions and were not related to QI were excluded.

This process narrowed the selection down to 75 publications deemed most pertinent for inclusion in the systematic review. The selected scientific articles were found to fall between 2004 and 2023. Although there was no time frame restriction, there were no further articles located prior to that time frame. This 19-year period provided a comprehensive overview of the developments and trends in neurosurgical quality control and improvement. During the screening stage, only articles written in the English language were considered, resulting in the exclusion of two non-English publications. This decision was made to maintain consistency and ensure that all reviewed articles were accessible to the research team.

## Results

**Figure 2:**
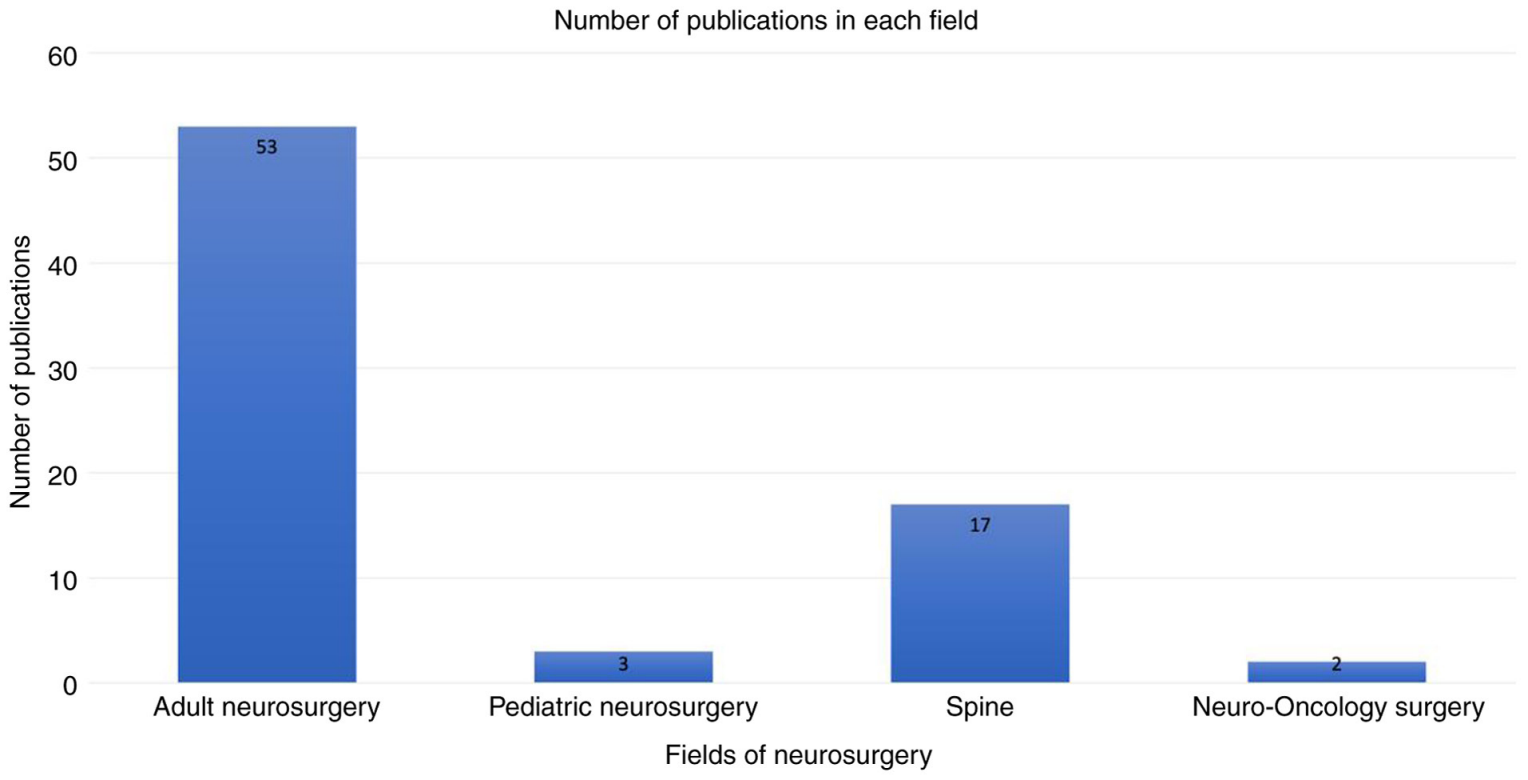
Bar chart depicting the number of quality improvement publications in four main fields in neurosurgery.

The 75 publications (listed in Table I) (2,4,5,10-81) revealed an uneven distribution across four main fields of neurosurgery. Adult neurosurgery encompassed the largest proportion of 70.5%, accounting for 53 articles (2,4,5,13,14,16-23,26-29,31-33,35-40,45,48,50-55,57-65,67-70,72-77). This was followed by spine surgery with 17 articles (22.5%) (10-12,24,30,41-44,46,47,56,71,78-81), pediatric neurosurgery with three articles (4%) (15,25,34) and neuro-oncology exclusive studies with the lowest proportion of 3%, representing two articles (49,66) (Table I and Fig. 2). There was also a marked disparity in the distribution of targeted patients (n=621,293) across the different fields of neurosurgery. QI studies focusing on spinal-only cases comprised the vast majority of patients at 87.07% (n=540,955) (10-12,24,30,41-44,46,47,56,71,78-81), while cranial-only studies and combined cranial and spinal studies accounted for 0.21% of the total patients collectively (n=1,309) (2,4,5,13,14,16-23,26-29,31-33,35-40,45,48,50-55,57-65,67-70,72-77). Additionally, the unspecified category accounted for 12.72% (73,29) of patients (15,25,34,49,66) (Table II).

**Figure 3:**
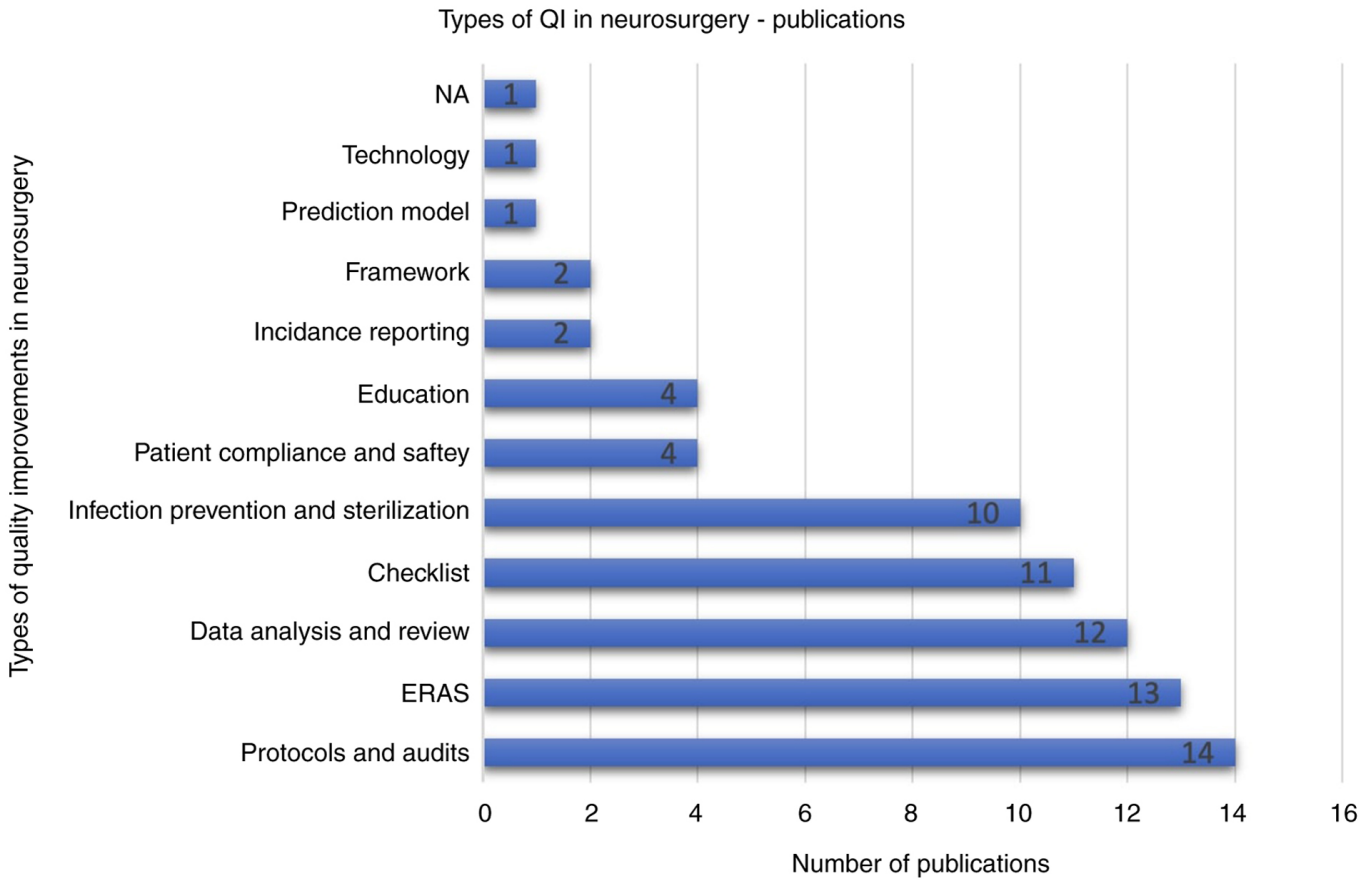
Bar chart depicting the different interventions used to provide quality improvement in neurosurgical care.

Different interventions were used to improve QI and enhance care in neurosurgery. Implementing new protocols and audits was the most common intervention with 14 articles (18.67%) (12,16,23,33,36,40,61,63,70,72-74,76,81). This was followed by enhanced recovery after surgery (ERAS) with 13 articles (17.33%) (27,39,42,49,57,71,80), data analysis of databases, registries, and literature with 12 articles (16%) (15,18,21,24-26,28,31,34,41,65,66), new or modified checklists implementation with 11 articles (14.67%) (2,19,32,35,37,50-52,54,55,79), and utilizing new sterilization devices or protocols with 10 articles (13.3%) (5,11,17,22,56,58,64,68,69,78). Less frequently addressed interventions were utilizing new technology, using a prediction model, improving incident reporting, increasing patient compliance, and educating the neurosurgical staff (10,13,14,20,29,30,38,53,59,60,75,77) (Fig. 3).

The study design varied within the 75 publications and covered the whole research design pyramid from systematic reviews, the most authentic and strongest research design, to case studies, the least authentic and weakest research design. The predominant design was systematic review studies with 24 articles (32%) (2,11,13,19,22,25,27,33,37,39,42,43,44,46,49,52,54,57-59,65-67,80), followed by randomized clinical trials with 11 articles (14.67%) (5,12,14,23,32,34,35,38,45,47,48), and prospective cohort studies with 10 articles (13.33%) (18,20,21,24,26,30,31,41,53,62). The least research designs used were prospective case-control studies with only one article (1.33%) (77), case reports with two articles (2.67%) (28,37), and correlation (10,29,36) and retrospective (40,64,71) studies with three articles (4%) each. Other types of studies, such as audit studies (4,16,17,61,63,73,74) and cross-sectional studies (15,50,51,56,60) were in between (Table III).

**Figure 4:**
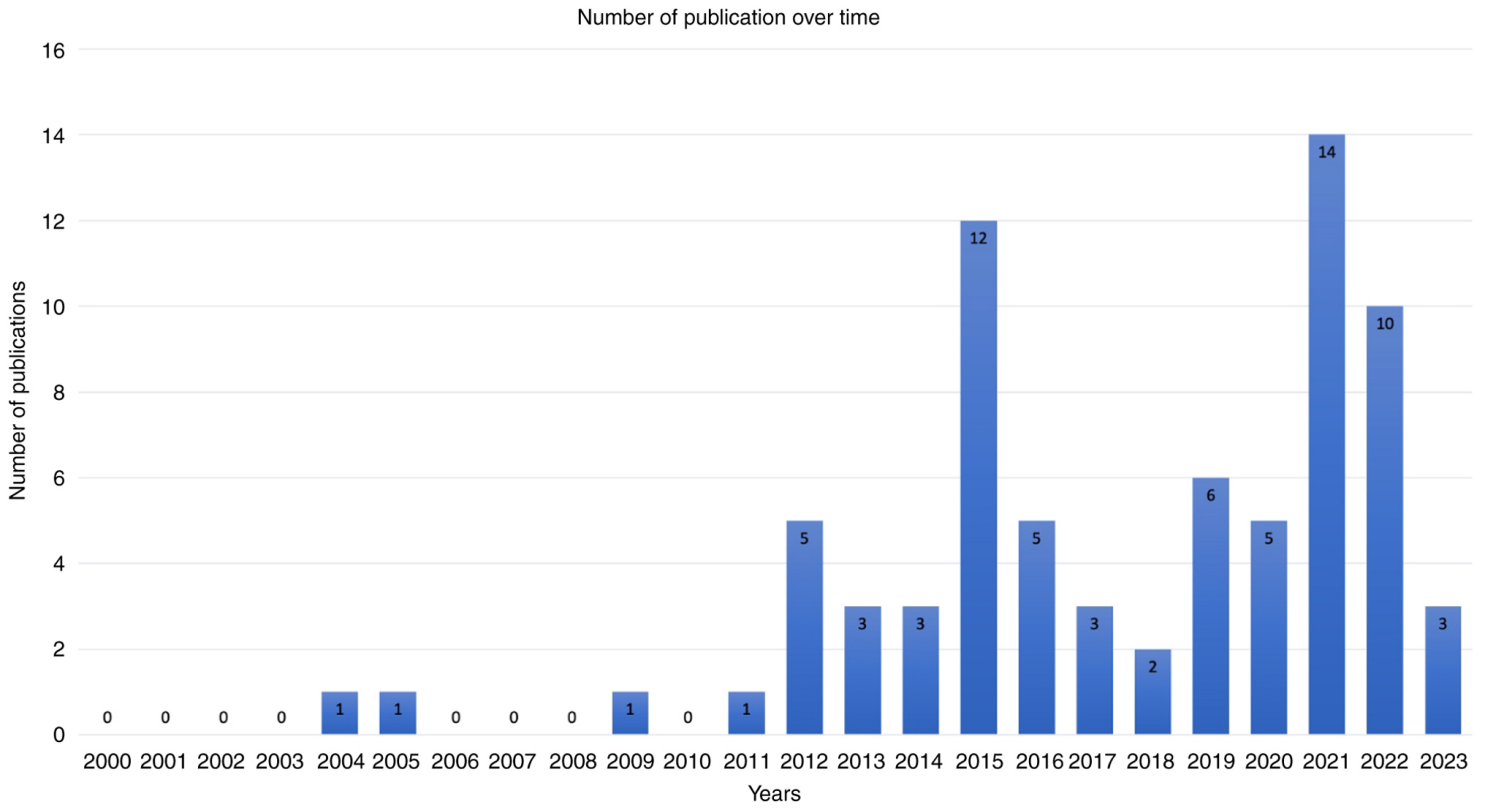
Bar chart depicting the number of publications in each year from 2000 to 2023.

**Figure 5:**
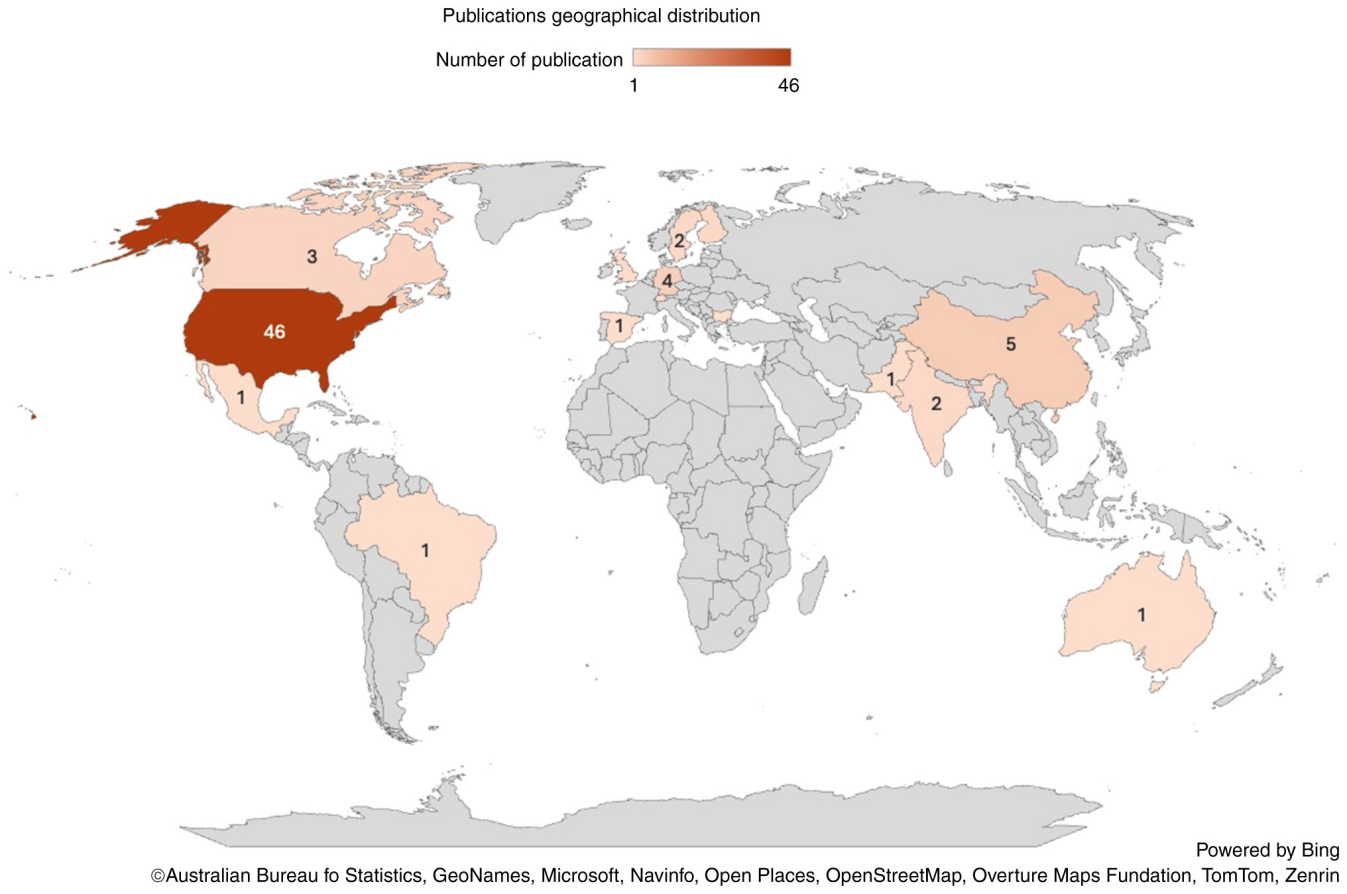
World map depicting the countries where quality improvement studies were published and their numbers.

The articles spanned through a period of 20 years from 2004 to 2023, with gaps of no publications in 2006-2008 and 2010. Notably, 81% of the publications were from 2012 to 2023 (2,4,5,10-23,25-59,61-66,68,81). The year 2021 had the highest number of publications with 14 articles (18.66%) (4,16,20,22,27,29,38,42,45-47,50,75,80), followed by 2015 with 12 articles (16%) (5,10,13,18,21,26,31,37,54,56,66,70), and 2022 with 10 articles (13.3%) (15,17,39,40,48,49,58,61-63). Conversely, 2004, 2005, 2009 and 2011 had the fewest publications, with only one article each (24,60,67,77), followed by 2018 with two articles each (14,71). The average number of articles per year between 2004 and 2023 was 3.75 (Fig. 4). Geographically, the distribution of publications was also uneven. The majority originated from the USA (46 articles) (4,5,10,11,13-15,18-31,33,34,38,40,41,43,47,49,53,56,61-63,65,66,68-73,75,76,78,81), and Germany and China with four and five articles, respectively (35,39,48,58,59,64,74,77,79). Contributions from other countries were fewer (Fig. 5).

## Discussion

QI in healthcare is crucial for various reasons: For enhancing the outcomes of patients, professional development, understanding healthcare challenges at local and national levels, and improving overall system performance. Given the inherent complexities and critical nature of neurosurgery, QI is exceptionally essential, rendering research in this area fundamental (26).

The results of the present study provide key insight into the QI initiatives within the field of neurosurgery. There is an uneven distribution of publications across the main subspecialties of neurosurgery: Adults, pediatrics, spine, cranial and neuro-oncology. This is similar to the findings of other studies and is due to the higher volume of procedures in one field more than the other and/or higher incidence of complications. The findings of the present study, similar to those of other research, also demonstrated a marked disparity in the distribution of targeted patients across the different fields of neurosurgery being more focused on spinal-only (82). The reason for this may be due to low morbidity rates and high efficacy in sustaining therapeutic outcomes of spinal surgery. As a result, this leads to an unintentional bias in the effort of QI research aimed to improve these outcomes and reduce post-operative complications (82). However, the limited attention given to other subspecialties, such as cranial procedures, is concerning. This imbalance is troubling as it may lead to disparities in the quality of care and patient outcomes across different neurosurgical fields. Future QI initiatives should aim to achieve a more equitable distribution of focus across all subspecialties to ensure comprehensive improvements in neurosurgical care (83).

The most common QI interventions are implementing new protocols, audits, ERAS, and data analysis from databases and registries. As was expected, it was found that these QI interventions reflected marked effectiveness in reducing operative complications and improving outcomes (84). However, other implementations may have a crucial impact on neurosurgical care and outcomes that have less QI research focus. For example, improving incident reporting, prediction models and new technologies. It may be beneficial for future studies to explore the impact of the less common interventions and to determine their impact across neurosurgical settings (6).

The findings presented herein highlight several key areas for future research and development in QI for neurosurgery specifically and healthcare in general. There is a need for more balanced attention across all neurosurgical fields particularly in areas, such as pediatrics and neuro-oncology. Expanding the diversity in QI interventions and exploring the efficacy of less common approaches will be crucial for developing comprehensive strategies, techniques and protocols that address the challenges of neurosurgical care.

The present study had some limitations, which should be mentioned. One of the notable limitations encountered during the study was the inability to access several articles due to paywalls. Despite efforts to obtain these publications, seven articles could not be accessed and were therefore excluded from the review. This limitation highlights a common challenge in academic research where financial barriers restrict access to potentially valuable information. Additionally, the scarcity of articles directly addressing the specific aims of this study posed another limitation. The targeted nature of the key words and the niche focus on neurosurgical quality control and improvement meant that there were relatively few articles available that fit the criteria precisely. As a result, it is possible that some relevant articles were not detected during the search process, potentially leading to an incomplete collection of data. This limitation underscores the importance of continued research and publication in this specialized area to build a more robust body of literature for future reviews.

In conclusion, QI studies enhanced care delivery for patients admitted to neurosurgery departments. The findings of the present study demonstrated that these studies were mainly focused on adult neurosurgery and primarily targeted patients who required spinal surgery. Furthermore, the most commonly employed methods to improve the quality of care include the implementation of new protocols, ERAS pathways, data analysis and new or modified checklists. Further research is required to bridge the gap by addressing QI initiatives in cranial surgery and evaluating the effectiveness of less-used modalities, such as new technologies and predictive models.

## Figures and Tables

**Table I:** Summary of all relevant findings in the included quality improvement studies.

Article no.	First author	Year of publication	Country	Field of neurosurgery	Targeted patients	No. of patients	QI intervention	Research design	(Refs.)
1	Ziewacz	2015	USA	Spine surgery	All patients undergoing neurosurgery	Unspecified	Education	Correlational study	(10)
2	Anderson	2016	USA	Spine surgery	Patients underwent spine surgery	Data on HAI from over 1545 hospital facilities with unspecified number of patients	Infection prevention and sterilization	Systematic review	(11)
3	Lindbäck	2017	Sweden	Spine surgery	Patients with degenerative lumbar spine disorder	197	Protocol and audit	Randomized clinical trial	(12)
4	McLaughlin	2015	USA	Adult neurosurgery	All neurosurgical procedures performed in the main operating room or the outpatient surgery center at the Ronald Reagan UCLA Medical Center and UCLA Santa Monica Medical Center from July 2008 to December 2012 were considered for this study except interventional radiology and stereotactic radiosurgery procedures	6,912	Incidence reporting	Systematic review	(13)
5	Robertson	2018	USA	Adult neurosurgery	Patients undergoing elective cranial or spinal neurosurgery	416	Patient compliance and safety	Randomized clinical trial	(14)
6	Pendola	2022	USA	Pediatric neurosurgery	Pediatric neurosurgery	129	Data analysis and review	Cross-sectional study	(15)
7	Ashraf	2021	Pakistan	Adult neurosurgery	Elective cases of both cranial and spinal neurosurgical diseases.	100	Protocol and audit	Audit study	(16)
8	Isaacs	2022	Canada	Adult neurosurgery	Adult patients (aged ≥18 years) undergoing VP shunt surgery	244	Infection prevention and sterilization	Audit study	(17)
9	Bekelis	2015	USA	Adult neurosurgery	Unspecified	Unspecified	Data analysis and review	Prospective cohort study	(18)
10	Zuckerman	2012	USA	Adult neurosurgery	All patients undergoing neurosurgical procedures.	Unspecified	Checklist	Systematic review	(19)
11	Ali	2021	USA	Adult neurosurgery	neurosurgical patients at an urban, level I trauma, academic teaching hospital.	Unspecified	Education	Prospective cohort study	(20)
12	Asher	2015	USA	Adult neurosurgery	Unspecified	Unspecified	Data analysis and review	Prospective cohort study	(21)
13	Kerezoudis	2021	USA	Adult neurosurgery	National Surgical Quality Improvement Program abstracted neurosurgical cases	317	Infection prevention and sterilization	Systematic review	(22)
14	Sisler	2017	USA	Adult neurosurgery	Patients 18 years of age or older who indicated current tobacco use (that is, in the 30 days prior to admission) were admitted to the inpatient neurosurgical service	526: 189 from the period between July 1, 2014, and December 31, 2014 taken as control and 337 from period between January 1, 2015 to December 31, 2015 taken as the intervention group	Protocol and audit	Randomized clinical trial	(23)
15	Deyo	2009	USA	Spine surgery	Patients underwent spine surgery	Unspecified	Data analysis and review	Prospective cohort studies	(24)
16	Ruiz Colón	2023	USA	Pediatric neurosurgery	Pediatric patients	Unspecified	Data analysis and review	Systematic review	(25)
17	Yang	2015	USA	Adult neurosurgery	Unspecified	Unspecified	Data analysis and review	Prospective cohort studies	(26)
18	Stumpo	2021	USA	Adult neurosurgery-cranial	Unspecified	Unspecified	ERAS	Systematic review	(27)
19	Wang	2020	USA	Adult neurosurgery	Unspecified	Unspecified	Data analysis and review	Case report	(28)
20	Meyrat	2021	USA	Adult neurosurgery	Ambulatory and hospital cases of neurosurgery	2,646: 2,270 were ambulatory dataset and 376 were a hospital dataset	Patient compliance and safety	Correlational study	(29)
21	Tanenbaum	2016	USA	Spine surgery	All adult patients aged 18 years and older included in the nation-wide inpatient sample (NIS) that underwent lumbar fusion from 1998-2011	53,9172	Patient compliance and safety	Prospective cohort study	(30)
22	Groman	2015	USA	Adult neurosurgery	Unspecified	Unspecified	Data analysis and review	Prospective cohort studies	(31)
23	Hall	2019	UK	Adult neurosurgery	Patients returning from the operating department to the neuro-surgical ward	100	Checklist	Randomized clinical trial	(32)
24	McLaughlin	2014	USA	Adult neurosurgery	All patients undergoing neurosurgery	Unspecified	Protocol and audit	Systematic review	(33)
25	Shi	2023	USA	Pediatric neurosurgery	Children undergoing spinal surgery	Unspecified	Data analysis and review	Randomized clinical trial	(34)
26	Oszvald	2012	Germany	Adult neurosurgery	All patients undergoing neurosurgery	12,390	Checklist	Randomized clinical trial	(35)
27	Sarnthein	2016	Switzerland	Adult neurosurgery-cranial	All patients undergoing neurosurgery	2,880	Protocol and audit	Correlational study	(36)
28	Zuckerman	2015	North America	Adult neurosurgery	All patients undergoing neurosurgery	Unspecified	Checklist	Systematic review	(37)
29	Ber	2021	USA	Adult neurosurgery	All patients undergoing neurosurgery	1,530	Technology	Randomized clinical trial	(38)
30	Liu	2022	China	Adult neurosurgery	Patients >65 years of age undergoing neurosurgeries	Unspecified	ERAS	Systematic review	(39)
31	Benjamin	2022	USA	Adult neurosurgery	Patients undergoing endoscopic endonasal resection of pituitary adenomas	171	Protocol and audit	Case-control study	(40)
32	Norris	2023	USA	Spine surgery	Patients ≥18 years of age meeting one of the following high-risk criteria: 8 + levels fused, osteoporosis with 4 + levels fused, three column osteotomy, anterior revision of the same lumbar level, or planned significant correction for severe myelopathy, scoliosis (>75˚), or kyphosis (>75˚)	263	Data analysis and review	Prospective cohort study	(41)
33	Debono	2021		Spine surgery	Patients undergoing lumbar spinal fusion	Unspecified	ERAS	Systematic review	(42)
34	Elsarrag	2019	USA	Spine surgery	Pediatric patients with spinal deformities	132	ERAS	Systematic review	(43)
35	Dietz	2019	India	Spine surgery	Patients with brain tumors	500	ERAS	Systematic review	(44)
36	Elayat	2021	India	Adult neurosurgery	Adult patients scheduled for elective supratentorial intracranial tumor excision	70	ERAS	Randomized clinical trial	(45)
37	Koucheki	2021	Canada	Spine surgery	Individuals with adolescent idiopathic scoliosis	2,456	ERAS	Systematic review	(46)
38	Kerolus	2021	USA	Spine surgery	Patients undergoing an elective single-level MIS TLIF for degenerative changes at a single institution	299	ERAS	Randomized clinical trial	(47)
39	Wang	2022	China	Adult neurosurgery	Patients who underwent elective craniotomy between January 2019 and June 2020.	151	ERAS	Randomized clinical trial	(48)
40	Greisman	2022	USA	Neuro-oncology	Patients undergoing cranial tumor resection.	Unspecified	ERAS	Systematic review	(49)
41	Suresh	2021	India	Adult neurosurgery	Undergoing elective neurosurgical procedures, specifically 131 cases of craniotomy and 69 cases of spine surgery	200	Checklist	Cross-sectional study	(50)
42	Westman	2020	Finland	Adult neurosurgery	Patients undergoing neurosurgical procedures.	Unspecified	Checklist	Systematic review	(2)
43	Lepänluoma	2014	Finland	Adult neurosurgery	Neurosurgical patients	150	Checklist	Cross-sectional study	(51)
44	Soriano Sánchez	2019	Mexico	Adult neurosurgery	Neurosurgical patients	Unspecified	Checklist	Systematic review	(52)
45	Lau.	2012	USA	Adult neurosurgery	Neurosurgical patients	Unspecified	Checklist	Prospective cohort study	(53)
46	Enchev	2015	Bulgaria	Adult neurosurgery	Neurosurgical patients	Unspecified	Checklist	Systematic review	(54)
47	Silva-Freitas	2012	Spain	Adult neurosurgery	Neurosurgical patients	400	Checklist	Pre/post-intervention study	(55)
48	Schaffzin	2015	USA	Spine surgery	Pediatric patients undergoing cardio-thoracic, neurosurgical shunt, and spinal fusion surgeries	Unspecified	Infection prevention and sterilization	Cross-sectional study	(56)
49	Pauli	2017	Brazil	Adult neurosurgery	Patients with mesial temporal lobe epilepsy undergoing anterior temporal lobectomy	50	ERAS	Systematic review	(57)
50	Tian	2022	China	Adult neurosurgery	Neurosurgical patients	24,137	Infection prevention and sterilization	Systematic review	(58)
51	Wang	2020	China	Adult neurosurgery	Patients admitted to the neurosurgery intensive care unit between January 2017 and February 2018, in Capital Medical University, Beijing, China	310	Prediction model	Systematic review	(59)
52	Annette	2005	Sweden	Adult neurosurgery	Neurosurgical intensive care unit patients	Unspecified	Education	Cross-sectional study	(60)
53	Krushelnytskyy	2022	USA	Adult neurosurgery	Neurosurgical patients	Unspecified	Protocol and audit	Audit study	(61)
54	Kassicieh	2022	USA	Adult neurosurgery-cranial	Patients with inter-hospital transfer status	47,736	NA	Prospective cohort study	(62)
55	Witiw	2015	USA	Adult neurosurgery	Neurosurgical patients	Unspecified	Infection prevention and sterilization	Randomized clinical trial	(5)
56	Neal	2021	USA	Adult neurosurgery	Neurosurgical patients	Unspecified	Framework	Audit study	(4)
57	Rotter	2022	USA	Adult neurosurgery	Patients requiring external ventricular drain (EVD) or intracranial pressure (ICP) monitor placement	Unspecified	Protocol and audit	Audit study	(63)
58	Schipmann	2016	Germany	Adult neurosurgery-cranial	Patients undergoing cranial neurosurgery	70 cases with surgical site infections and 185 matched controls	Infection prevention and sterilization	Case-control studies	(64)
59	Rubiano	2012	USA	Adult neurosurgery-cranial	Neurotrauma patients in low- and middle-income countries	Unspecified	Data analysis and review	Systematic review	(65)
60	Fischer	2015	USA	Neuro-oncology	Pediatric patients with brain tumors	Unspecified	Data analysis and review	Systematic review	(66)
61	Bernstein	2004	Canada	Adult neurosurgery	Patients undergoing novel neurosurgical procedures	Unspecified	Framework	Systematic review	(67)
62	Leming-Lee	2019	USA	Adult neurosurgery	Neurosurgical patients undergoing craniotomy procedures	Unspecified	Infection prevention and sterilization	Pre/post-intervention studies	(68)
63	Hover	2013	USA	Adult neurosurgery	Patients undergoing elective neurosurgical procedures	Unspecified	Infection prevention and sterilization	Pre/post-intervention studies	(69)
64	Mathews	2015	USA	Adult neurosurgery	Elective neurosurgical patients	2,328	Protocol and audit	Case report	(70)
65	Rupich	2018	USA	Spine surgery	Postoperative neuro-surgical spine patients	Unspecified	ERAS	Case-control study	(71)
66	Rozman	2020	USA	Adult neurosurgery	Neurosurgical patients	Unspecified	Protocol and audit	Pre/post-intervention study	(72)
67	Farrokhi	2013	USA	Adult neurosurgery	Patients undergoing minimally invasive spine surgery	Unspecified	Protocol and audit	Audit study	(73)
68	Xu	2013	China	Adult neurosurgery	Critically ill patients in a neurosurgical intensive care unit	Unspecified	Protocol and audit	Audit study	(74)
69	Chang	2021	USA	Adult neurosurgery-cranial	Patients requiring external ventricular drain (EVD) placement in the emergency department	38 (20 during protocol initia tion and 18 pre-protocol)	Patient compliance and safety	Pre/post-intervention study	(75)
70	Ezeamuzie	2019	USA	Adult neurosurgery	Patients undergoing complex surgical procedures with increased operative time)	212	Protocol and audit	Prospective case-control study	(76)
71	Kantelhardt	2011	Germany	Adult neurosurgery	Neurosurgical patients	Unspecified	Incidence reporting	Pre/post-intervention studies	(77)
72	Ryan	2014	USA	Spine surgery	Pediatric patients undergoing complex spine surgery	267	Infection prevention and sterilization	Pre/post-intervention study	(78)
73	Kantelhardt	2016	Germany	Spine surgery	Patients undergoing spinal surgery	149	Checklist	Pre/post-intervention study	(79)
74	Licina	2021	Australia	Spine surgery	Patients undergoing spinal surgery	Unspecified	ERAS	Systematic review	(80)
75	Young	2020	USA	Spine surgery	Patients undergoing elective spine procedures	1,000	Protocol and audit	Pre/post-intervention study	(81)

**Table II:** Quality improvement in neurosurgeries and sub-subspecialties.

Targeted patients	No. of patients	Percentage	(Refs.)
Spinal only	540,955	87.07	(10-12,24,30,41-44,46,47,56,71,78-81)
Unspecified	79,029	12.72	(15,25,34,49,66)
Cranial + spinal	673	0.11	(13,14,16-23,25,26,28,29,31-33,35,37-40,45,48,50-55,57-4,5,^61^,^63^,^67-70^,^72-74^,^76^,^77^)
Cranial only	636	0.1	(27,36,62,64,65,75)

**Table III:** Research design of the selected studies.

Types of studies	No. of studies	Percentage	(Refs.)
Systematic review studies	24	32.00	(2,11,13,19,22,25,27,33,37,39,42,43,44,46,49,52,54,57-59,65,69,67,80)
Randomized clinical trials	11	14.67	(12,14,23,32,34,35,38,45,47,48,63)
Prospective cohort studies	10	13.33	(18,20,21,24,26,30,31,41,53,62)
Pre/post-intervention studies	9	12.00	(55,68,69,72,75,77,78,79,81)
Audit studies	7	9.33	(4,16,17,61,63,73,74)
Cross-sectional studies	5	6.67	(15,50,52,56,60)
Case-control studies	3	4.00	(40,64,71)
Correlation studies	3	4.00	(10,29,36)
Case reports	2	2.67	(28,37)
Prospective case-control studies	1	1.33	(76)

## Data Availability

The data generated in the present study may be requested from the corresponding author.
